# The development path of the medical profession in China’s engineering universities from the perspective of the ‘four new’ disciplines

**DOI:** 10.1080/07853890.2022.2139409

**Published:** 2022-10-29

**Authors:** Li Yan, Huijing Hu, Yu Zheng, Yin Zhou, Le Li

**Affiliations:** a Institute of Medical Research, Northwestern Polytechnical University, Xi’an, China; b Department of Ultrasonography, Xían Central Hospital, Xi’an, China

**Keywords:** Medical education, medical engineering, interdisciplinary, integrated medicine, composite curriculum, graduate student, undergraduates

## Abstract

In recent years, China has actively promoted the construction of first-class universities and disciplines of the world (‘Double First-Class’), and built a new model of university development to solve Chinese problems and support high-quality economic and social development. In the context of China’s efforts to promote the construction of new engineering, new medicine, new agriculture, and new liberal arts (referred to as the ‘four new’ disciplines), these disciplines are developing rapidly. As a specialty dealing with major life issues, medical education has become increasingly prominent. To enhance the comprehensive strength of universities, corresponding to the ‘four new’ disciplines strategy, engineering universities are building and developing medical specialties one after another. At present, the greatest problem in the medical specialty of engineering universities is the tendency to blindly follow trends and integrate new concepts with traditional methods. However, to date, the integration of medical and nonmedical specialties has been superficial and thus has not been successful. To address this problem, this paper, guided by the policies aimed at developing the ‘four new’ disciplines, analyses the current situation of traditional medicine education and professional development in engineering universities and proposes measures to enhance the competitiveness of new medicine in engineering universities, thereby promoting the development of universities.

**KEY MESSAGES**
- The implementation of the ‘four new’ disciplines is a strategic choice for higher education.
- Engineering technology is an efficient path and hands-on approach to solving medical problems.
- Interdisciplinary and comprehensive educational approaches play an important role in the development of medical science.

## Introduction

In the context of China’s efforts to create first-class universities and disciplines of the world (‘Double First-Class’), the development of medical education is an important way to promote the development of universities [1]. High-level medical education has become an important aspect of higher education and the fostering of a ‘Double First-Class’. In October 2018, to accelerate the development of high-level undergraduate education and comprehensively improve efforts to cultivate academic talent, the Ministry of Education of the People’s Republic of China issued the *Opinions on Accelerating the Construction of High-level Undergraduate Education and Comprehensively Improving the Ability of Talent Training* and other documents and implemented the *Excellent Engineer Education and Training Programme 2.0, Excellent Doctor Education and Training Programme 2.0, Excellent Agriculture and Forestry Talent Education and Training Programme 2.0, Excellent Teacher Training Programme 2.0, Excellent Legal Talent Education and Training Programme 2.0, Excellent Journalism and Communication Talent Education and Training Programme 2.0 and Training of Top Students in Basic Disciplines Plan 2.0* (‘Six Excellences and One Top-notch’ plan 2.0) [2]. These directives sought to promote the construction of new engineering, new medicine, new agriculture, and new liberal arts (referred to as the ‘four new’ disciplines); improve the ability of universities to support social development; and achieve the intensive development of higher education [3].

The construction of the ‘Double First-Class’ is a major strategic decision of the Central Committee of the Communist Party of China and the State Council, and it is a leading and landmark project for the construction of higher education for a powerful country in the new era, designed to enhance the comprehensive strength and international competitiveness of higher education in China [4]. The construction of the ‘four new’ disciplines is the goal of China’s higher education reform, not only to meet the external requirements of the development of higher medical education in the new era but also to follow the ‘internal logic’ of medical discipline development. Additionally, it is intended to meet the requirements of the new round of scientific and technological revolution and industrial reform. ‘New engineering’ is an attempt to actively deal with the fourth industrial revolution and enhance national hard power, ‘new medicine’ is an important foundation for building a healthy China and promoting the health of all people, ‘new agriculture’ is an important measure for implementing the concept of an ecological civilization and enhancing ecological growth, and ‘new liberal arts’ is an important carrier to develop socialist advanced culture and enhance the soft power of national culture. In the process of the development of the ‘four new’ disciplines, they are intertwined and support each other: ‘You have me, and I have you’. The development of ‘new medicine’ and the reform of medical education must rely on the power and promote the trends of new engineering, new agriculture and new liberal arts [5,6].

Medical education should be based on the principles of complementary advantages, resource sharing, the integration of medical teaching and practice, and win–win cooperation, and the existing advantages should be utilized to enhance interdisciplinary integration, i.e. medicine + engineering, medicine + science, and medicine + liberal arts [7]. Moreover, new modes of personnel training, scientific research, and transformation of scientific and technological achievements should be explored; advanced medical care should be practised; useful medical education experience should be integrated and high-end medical talent recruited from countries all over the world; and cooperation among government departments, hospitals, and medical-related enterprises and institutions should be strengthened [8–10]. For example, new engineering and new medicine programmes launched by universities can be combined to break down barriers between traditional disciplines and colleges and cultivate talent with interdisciplinary (both medical and engineering) skills [11]. The medical personnel training mode has been reformed, and this school–enterprise collaboration supports the development of multilevel and multidimensional cooperation between schools and enterprises, thus strengthening practical teaching and improving the development of practical skills among students—as well as their analytical and problem-solving abilities and innovativeness—and is therefore of great practical value for cultivating competent and applied talent to serve the regional economy [6].

In universities with distinct engineering characteristics, scientific problems in the field of medicine are both the introduction to and targets of engineering technology, and engineering technology is the best path and hands-on approach to solving medical problems. Therefore, an interdisciplinary and comprehensive educational approach is important in the future development of medical science [12]. To meet the needs of the current era and cultivate talent suitable for rapid research and development of medical equipment and software, it is necessary to optimally integrate medicine with engineering, materials science, and computer science and to train students to closely integrate leading scientific and technological problems in clinical practice with cutting-edge technologies in engineering [13]. In terms of evaluating the Programme for training talent, the only important criterion is whether students can truly obtain interdisciplinary skills and have sufficient ability to use engineering methods when dealing with medical problems [14].

At present, the greatest problem in the medical specialty of engineering universities is the tendency to blindly follow trends and integrate new concepts with traditional methods [3]. However, to date, the integration of medical and nonmedical specialties has been superficial and thus has not been successful [15]. To address this problem, this paper, guided by the policies aimed at developing the ‘four new’ disciplines, analyses the current situation of traditional medicine education and professional development in engineering universities and proposes measures to enhance the competitiveness of new medicine in engineering universities, thereby promoting the development of universities.

## Discussion

**Figure 1.**
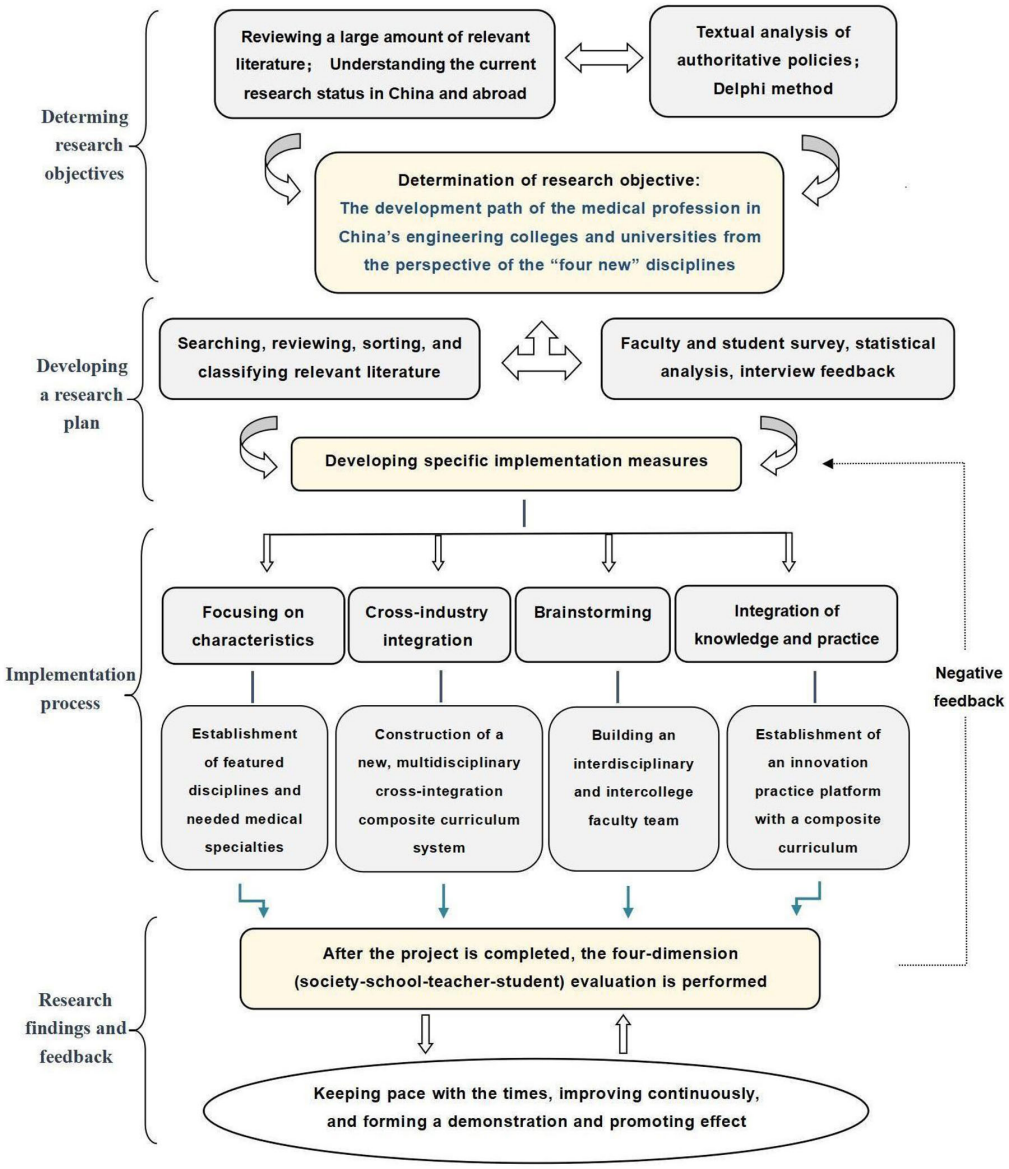
The framework for the development of new medicine in engineering universities.

This paper is based on engineering universities in Shaanxi Province that have a medical institute/medical specialty. With the school as the carrier and all teachers and students as the research object, information was obtained from the universities through questionnaires, interviews and consultation with authoritative experts to explore the path of the integrated development of medical education (Figure 1).

### Breaking down barriers between disciplines

New medicine adapts to the requirements of new scientific and technological advances and industrial transformation; proposes a new concept of lifelong and holistic medicine, which changes the conception of health care from a treatment-oriented approach to one of preventive treatment and health preservation; and establishes new fields of precision medicine, translational medicine, and intelligent medicine [16]. Under the ‘four new’ disciplines perspective, interdisciplinary integration needs to be further deepened. For example, traditional medicine needs to be further integrated with new technologies, such as artificial intelligence, new materials, and big data [17].

Taking the fields of aeronautics, aerospace, and navigation as an example, the medical profession should study and attempt to solve various medical problems that arise in the special environments of aviation, aerospace, navigation, and underwater exploration to ensure the safety, health, comfort, and working ability of operators and professionals in these environments [18]. The changes in cardiovascular function and circulatory system regulation caused by space flight directly affect the health and work performance of astronauts, with weightlessness being the most important environmental factor [19]. At present, researchers have some experience with the physiological changes and adaptation patterns of the human body during short-term aerospace flight, but their understanding of the patterns and characteristics of cardiovascular changes caused by long-term weightlessness and the mechanisms underlying the related physiological effects is not comprehensive, and the corresponding cardiovascular protection measures still need to be improved [20]. Clarifying and solving these problems is an urgent need for China’s manned spaceflight strategy.

The medical humanities have emerged in many parts of the world as an increasingly popular teaching modality in medical education [21]. As for arts and humanities, it is reported that they have tremendous diversity and potential in medical education as intrinsic to, additive to or curative for medicine [22]. To solve such real-world problems, medical students must have national interests in mind; grasp the necessary interdisciplinary knowledge; actively integrate multiple disciplines into their practical coursework; apply their studies to actual industry projects, scientific research projects, and topics for guidance; and receive innovative entrepreneurial training in a project-guided teaching mode.

### Cross-industry integration and construction of a new composite curriculum system

In line with the concept of complementary advantages and win–win cooperation, the medical needs of other majors in universities should be actively investigated so that the curriculum system can be reconstructed on the basis of existing training programmes [23]. These related disciplines cannot be separated from the medical profession, and medical knowledge can be integrated into the training of nonmedical professionals. In the curriculum setting and in constructing a knowledge system, simple module-based training should be avoided, and an innovative ‘double-track’ integrated training model should be explored [10]. In this model, the context of two main lines of study is clear, but these disciplines are integrated with each other. In different periods of study, the primary and secondary lines are distinct, forming a new, staged professional curriculum system with multipoint cross-integration.

First, biomedical engineering and the frontiers of medical development should be introduced in the freshman and sophomore years. Teachers with backgrounds in clinical medicine, basic medicine, biomedical engineering, engineering, and other sciences could teach medical knowledge from different disciplinary perspectives to broaden the knowledge of medical students so that they can adapt to the concept of integrated medicine upon admission. Second, in junior and senior professional curricula and elective courses, cross-integrated courses (Med + X), such as medicine and NBIC (nanotechnology, biotechnology, information technology, and cognition) should be set up to enhance other professional skills in the medical curriculum [24]. Interdisciplinary lecture preparation can be adopted to link medicine-related disciplines to maximize their contribution to the teaching of important concepts [25].

### Brainstorming to build an interdisciplinary faculty team

Cross-disciplinary training is not only cross-disciplinary, cross-industry, and cross-professional but also cross-temporal and cross-cultural [10]. For example, in intelligent medicine, new medical talent must have not only the knowledge and skills of traditional medicine but also those of multiple fields, such as artificial intelligence, big data, electronic science and technology, computers, and the internet so that they become familiar with different areas of professional knowledge (liberal arts and sciences), learn to coordinate curriculum and ideology, and can solve practical problems that they will face in the field [26].

Taking medicine + materials science as an example, interdisciplinary and interschool teams of experts in inorganic biomaterials, organic biomaterials, composite biomaterials, and hybrid biomaterials can take advantage of the hierarchical, multicategory innovation and practice platform provided by scientific research teams in disciplines such as materials science, clinical medicine, and mechanics [10]. Discussions should be regularly conducted with medical institutions and biomedical materials companies on technical needs, product needs, and the necessary knowledge and skills of practitioners, and multidisciplinary integration should be promoted in the transition from interdisciplinary integration to science integration. Through the open experimental platforms of different disciplines, a large number of senior doctors and the technical experts of nonmedical companies can be invited to join the teaching team to cultivate compound talent.

### Integrating knowledge and practice and establishing a practice platform

In the process of practical teaching, the new trend of multidisciplinary collaboration and integration required in the training of innovative talent could be pursued to establish a platform that combines medical materials; medicine for the fields of aeronautics, aerospace, and navigation; and intelligent medicine [5]. The progressive training model of ‘learning during practice – training in innovative thinking’, ‘researching during learning – practising innovative methods’ and ‘innovating during researching – enhancement of innovativeness’ can be implemented for students at different levels [10]. By refining the system to recognize credits for courses cultivating innovation, ensuring funding for training in innovation, and formulating an incentive system for instructors to teach innovative practices, an open, cross-disciplinary platform can be developed that leverages scientific research teams to integrate innovation with professional practice. At the same time, attention should be paid to the cultivation of students’ interest in scientific research. There is a saying that ‘people who love it are better than those who know it, and people who delight in it are better than those who love it’ [27]. Taking joy in learning not only improves learning efficiency but also deepens students’ understanding of the material, allowing them to more effectively apply what they have learned [28].

Through the two major channels of practical teaching and innovation activities, innovative ability can be cultivated in a cross-curricular system. The practical teaching channel includes the three links of professional curriculum design, comprehensive training in professional experimental methods, and providing the knowledge necessary for graduation [11]. Relying on scientific research cooperation between medicine and other disciplines, professional knowledge is applied in the form of topics and projects. At the same time, attention should be paid to interaction with other systems, such as new engineering, in order to establish a broad medical platform and comprehensively integrate new and emerging medical fields, such as precision medicine and translational medicine. Additionally, high-level medical innovators who can adapt to the new generation of technological advances represented by artificial intelligence and changes in the life sciences represented by synthetic biology and who can apply interdisciplinary knowledge to solve frontier problems in the medical field should be cultivated [29].

With the increasingly urgent demand for high-level innovative talent in China, against the background of the new era, talent training and scientific and technological innovation are becoming increasingly closely integrated, and discipline construction and personnel training must be promoted by knowledge production and innovation. The construction of the ‘four new’ disciplines is gradually breaking down boundaries of undergraduate education and becoming an important measure to ‘build first-class majors and train first-class talents’, especially to cultivate high-level innovative talent [23]. In January 2022, the Ministry of Education and the Ministry of Finance, in ‘Some opinions of the National Development and Reform Commission on further promoting the construction of world-class universities and first-class disciplines’, proposed supporting the construction of universities aiming at the frontiers of world science and key technological fields to optimize discipline organization, integrate traditional discipline resources, and strengthen the discipline foundations of personnel training and scientific and technological innovation. In addition, existing discipline systems should be adjusted and upgraded, the barriers between disciplines and specialties should be eliminated, the construction of new engineering, new medicine, new agriculture and new liberal arts should be promoted, there should be an active response to the social demand for high-level talent, interdisciplinary specialties should be developed, and discipline growth points should be cultivated [4]. Thus, the construction of the ‘four new’ disciplines against the new background has become a practical process of exploring contact points between different disciplines, cultivating discipline growth points, and perfecting and even reconstructing the knowledge system (Table 1).

**Table 1.** Integration of four new disciplines into medical education.

	New engineering	New medicine	New agriculture	New liberal arts
Function and goal	Enhance national hard power	Improve national health	Enhance ecological growth	Enhance cultural soft power
How it is related to medicine	Aim at the frontier of medicine-engineering interdisciplinary integration, carry out in-depth cooperation in medicine-engineering integration in the fields of rehabilitation aids, smart medicine and health, biomechanics, new biomaterials, etc.	Move medical education forwards and backwards from the area of treatment, that is, from the original treatment to the trinity of prevention, treatment and health care, from treatment to the whole life cycle and the whole process of health (new concept) [30].	Promote the intersection of biology, biotechnology and other disciplines with forestry engineering and forestry majors, transform and upgrade existing agriculture-related majors, and deploy new industries such as intelligent agriculture, agricultural big data, forest health care, and ecological restoration.	Strengthen the cross-integration of medicine and humanities, promote the organic integration of humanities education and medical education, and cultivate medical talents with ‘temperature’ and ‘depth’ [31].
Advantages	Realize the shift from engineering orientation to industry demand orientation, from professional segmentation to cross-border cross-integration, and from adaptive services to support and leadership.	Focus training on grasping the five skills: Taoism, benevolence, academics, technology and art (new connotation).	Face new agriculture, new villages, new farmers, and new ecology, and promote the construction of new agricultural sciences to further deepen and improve to promote the comprehensive revitalization of rural areas and improve the quality of life and empowerment of the people.	The construction of new liberal arts belongs not only to China but also to the world; it not only plays a leading role in China’s development but also has a Chinese school, Chinese voice, and Chinese appeal and influence in shaping power in the development of the world.
Challenges	At present, there is a shortage of senior engineers with a medical interdisciplinary background, but it is difficult to encourage most medical or engineering discipline leaders to break through knowledge barriers and carry out interdisciplinary communication and research [32].	The construction of ‘new medicine’ corresponds to the requirements of the new round of the scientific and technological revolution and industrial reform, and realizes the integration of traditional disciplines and specialties with artificial intelligence, big data, robotics and other technologies.	Efforts should be made to enhance the ability to train new talents who know and love farmers, to enhance the level of agricultural scientific and technological innovation, to enhance the contribution to serving ‘agriculture, rural areas and farmers’, and to enhance the international competitiveness of agricultural and forestry education.	To enhance cultural soft power, the construction of new liberal arts corresponds to the reorganization of disciplines and the intersection of arts and sciences compared with traditional liberal arts, that is, the integration of new technologies into courses such as philosophy, literature, and language [33].
Representative specialty	Biomedical engineering, intelligent medical engineering, medical imaging technology and application, etc.	Precision medicine, translational medicine, intelligent medicine, etc.	Veterinary medicine, biology, etc.	Medical humanities English, medical insurance, etc.
Construction requirements	New specialties, new requirements, cross-integration and innovation are where the ‘new’ lies. The ‘four new’ disciplines are a new educational reform in the new era, a new quality of Chinese higher education in the new era, a new system of higher education in the new era, a new quality culture of universities in the new era, and a high-quality revolutionary project.			

As a trend of medical education, the cross-professional education model is closely related to the expansion of the scope of medical services and the actual needs of talent training [16]. Most major medical innovations have occurred in interdisciplinary fields, such as research and development of various medical imaging equipment and medical materials, and these innovations are inseparable from interdisciplinary cooperation and communication in fields such as physics and engineering [29,34]. In today’s rapid development of science and technology, it is insufficient to rely on a certain discipline to promote the development of human society. Therefore, to further promote the development of science and technology, to serve humanity, to realize the new medicine proposed by the Ministry of Education, and to transform the treatment-oriented approach into an approach that integrates diagnosis and treatment and intelligent medicine, it is necessary to promote the deep integration of engineering technology and medical technology.

In recent years, against the background of profound changes in global economic and social development, to train the talent needed today and in the future, the United States, Britain, Japan, Germany and other developed countries have been actively seeking a new round of higher education reform. To gain the strategic high ground in the new round of scientific and technological revolution and industrial reform and to contribute to jointly creating a new international model of higher education, China is actively planning and vigorously promoting the ‘four new’ discipline reforms in the field of higher education [35]. Medicine is multidisciplinary and interdisciplinary, and personnel training is a very complex educational system in engineering. In the ‘four new’ disciplines, the training mode of medical professionals with engineering colleges as the background has some limitations but also has certain advantages. Although we have made many efforts and explorations to innovate personnel training in this field, due to differences in educational systems and training models in different countries, the wide applicability of these developments needs to be further investigated, practised and improved.

## Challenges

For our programme, the most important thing is to invigorate the medical specialty and let it maximize ‘glow’ and ‘fever’ in engineering universities, achieve ‘scattered focus’, and focus on connotative development. Medical education is complex in nature and faces many challenges. It needs more understanding and support from the government and the public. Those who understand medicine have the responsibility and the right to pursue their own politics in their positions [36]. Only with more focussed input and dedication can they keep up with development, promote development and make due contributions.

## Conclusions

Currently, medical education is at a crossroads. Facing the challenges associated with scientific, technological, and social changes, the medical profession is becoming increasingly outdated as it faces new requirements for teaching content and social practices. Due to various factors, including deep-rooted values, natural resistance to change, and the certification process, the road to major curriculum reform is difficult [37,38]. *Rethinking Education: Towards a global common good?*, issued by the United Nations Educational, Scientific and Cultural Organization (UNESCO) in 2015, pointed out that given the current rapid development of science and technology and the challenges of globalization, the world is changing, and education must change with the times [39,40]. In the face of the new trends, new fields, and new directions represented by the scientific and technological revolution and the industrial revolution, medical education cannot remain apart and ‘independent’, and profound changes should be adopted to explore a new development path aligned with the trend of the times and the changing scientific and social context.

In this situation, the establishment of a new composite medical curriculum system, the establishment of a multilevel and multicategory innovative practice platform, the construction of cross-disciplinary and cross-school faculty teams, and the enhancement of students’ innovativeness based on the cross-integration of medicine and other disciplines could be the main strategy for training innovative talent in the new medicine. Against the background of the ‘four new’ disciplines, universities need to further revitalize their existing resources, focus on intensive development, and promote the transition from interdisciplinary integration to science integration. Universities should vigorously develop emerging disciplines, expand new growth points, enhance their comprehensive competitiveness, and accelerate their development as world-class institutions [41].

Finally, future medical education must become a modern system that pays attention to the cultivation of comprehensive quality. Medical education should emphasize a multidisciplinary approach, not only in natural sciences but also in humanities, engineering and agronomy, to better contribute to the medical cause and serve humankind.

## Data Availability

Data sharing is not applicable to this article as no new data were created or analysed in this study.
